# Role of treatment on the development of secondary malignancies in patients with essential thrombocythemia

**DOI:** 10.1002/cam4.1081

**Published:** 2017-05-23

**Authors:** Cristina Santoro, Isabella Sperduti, Roberto Latagliata, Erminia Baldacci, Barbara Anaclerico, Giuseppe Avvisati, Massimo Breccia, Francesco Buccisano, Michele Cedrone, Giuseppe Cimino, Cinzia De Gregoris, Marianna De Muro, Ambra Di Veroli, Sabrina Leonetti Crescenzi, Marco Montanaro, Enrico Montefusco, Raffaele Porrini, Angela Rago, Antonio Spadea, Francesca Spirito, Nicoletta Villivà, Alesssandro Andriani, Giuliana Alimena, Maria Gabriella Mazzucconi

**Affiliations:** ^1^ Hematology, Department of Cellular Biotechnologies and HematologySapienza University of RomeRomeItaly; ^2^ Biostatistical UnitRegina Elena National Cancer InstituteRomeItaly; ^3^ HematologySan Giovanni HospitalRomeItaly; ^4^ HematologyUniversity Campus BiomedicoRomeItaly; ^5^ HematologyUniversity Tor VergataRomeItaly; ^6^ HematologyPolo Universitario PontinoLatinaItaly; ^7^ HematologyBelcolle HospitalViterboItaly; ^8^ HematologySandro Pertini HospitalRomeItaly; ^9^ HematologySant'Andrea HospitalRomeItaly; ^10^ Hematology and Stem Cell Transplant UnitRegina Elena National Cancer UnitRomeItaly; ^11^ HematologySan Camillo HospitalRomeItaly; ^12^ HematologyNuovo Regina Margherita Hospital ASL Roma 1RomeItaly

**Keywords:** Alkylating agents, alpha‐interferon, anagrelide, essential thrombocythemia, hydroxyurea, secondary malignancy

## Abstract

Aim of this study is to explore the role of different treatments on the development of secondary malignancies (SMs) in a large cohort of essential thrombocythemia (ET) patients. We report the experience of a regional cooperative group in a real‐life cohort of 1026 patients with ET. We divided our population into five different groups: group 0, no treatment; group 1, hydroxyurea (HU); group 2, alkylating agents (ALK); group 3, ALK + HU sequentially or in combination; and group 4, anagrelide (ANA) and/or *α*‐interferon (IFN) only. Patients from groups 1, 2, and 3 could also have been treated either with ANA and/or IFN in their medical history, considering these drugs not to have an additional cytotoxic potential. In all, 63 of the 1026 patients (6%) developed 64 SM during the follow‐up, after a median time of 50 months (range: 2–158) from diagnosis. In univariate analysis, a statistically significant difference was found only for gender (*P* = 0.035) and age (*P* = 0.0001). In multivariate analysis, a statistically significant difference was maintained for both gender and age (gender HR1.7 [CI 95% 1.037–2.818] *P* = 0.035; age HR 4.190 [CI 95% 2.308–7.607] *P* = 0.0001). The impact of different treatments on SMs development was not statistically significant. In our series of 1026 ET patients, diagnosed and followed during a 30‐year period, the different therapies administered, comprising HU and ALK, do not appear to have impacted on the development of SM. A similar rate of SMs was observed also in untreated patients. The only two variables which showed a statistical significance were male gender and age >60 years.

## Introduction

Essential thrombocythemia (ET) is a myeloproliferative neoplasm, mostly occurring in elderly patients, frequently associated with major and minor vascular complications that cause increased morbidity and sometimes fatal complications 1, 2, 3. Survival of ET patients during the first 10 years of the disease is similar to that of the general population of the same age. In fact, life expectation is longer than 10 years in 80–95% with the most frequent causes of death being represented by thrombohemorrhagic events and secondary malignancies 4, 5, 6.

Cytoreduction is indicated in patients above 60 years of age or in those with previous thrombosis or a platelet count in excess of 1500 × 10^9^/L 7, 8. In these cases, hydroxyurea has emerged as the treatment of choice on account of its efficacy in reducing thrombotic complications 9. Other cytoreductive and/or immune modulating drugs, including alkylating agents, *α*‐interferon, and anagrelide, have been used and are still indicated in high‐risk patients 7, 8.

Several reports however have raised concerns about the long‐term safety of hydroxyurea and alkylating agents 10, 11, 12, 13. Although it is well known that these latter (given alone or in combination/sequentially) increase the risk of transformation into acute myeloid leukemia (AML), the specific role of different therapies on the development of secondary malignancies (SMs) in ET patients is still under investigation 14, 15, 16, 17, 18.

On the other hand, it has been hypothesized that ET has the intrinsic potential to evolve into malignant diseases, also because of the presence of an impaired “tumor immune surveillance,” low‐grade chronic inflammatory state, accumulation of reactive oxygen species (ROS), and favorable role of platelets to promote cellular growth, angiogenesis, and metastasis 19, 20.

Taking into account all the above‐mentioned considerations and uncertainties, we focused our study on ET treatment and SMs, with the aim to retrospectively evaluate the possible role of different therapies on the development of SMs in a large cohort of ET patients diagnosed and followed during a 30‐year period.

## Patients and Methods

In total, 11 hematologic centers (universities 5, general hospitals 6) from the region Lazio in Central Italy were involved in the study. To be included in the analysis, patients had to have received a diagnosis of ET, according either to Polycythemia Vera Study Group (PVSG) or World Health Organization (WHO) criteria 21, 22, 23, performed in a period between 1980 and 2010.

For data analysis, we did not consider the development of acute leukemia (AL) as a SM because this event could be considered as part of the natural evolution of ET; we also excluded the basaliomas because they cannot be considered real neoplasms and because it is well known that the use of hydroxyurea can cause this kind of skin tumors. On the other hand, we did not exclude squamous cell carcinomas because, in some instances, they could be relatively aggressive with local infiltration.

Taking in consideration the different treatment approaches, we divided our population in five different groups: group 0, no treated patients; group 1, patients treated with hydroxyurea (HU); group 2, patients treated with alkylating agents (mostly pipobroman, very few patients treated with melphalan) (ALK); group 3, patients treated with ALK + HU sequentially or in combination; and group 4, patients treated with anagrelide (ANA) and/or *α*‐interferon (IFN) only.

Patients from groups 1, 2, and 3 could also have been treated either with ANA and/or IFN in their medical history, considering these drugs not to have an additional cytotoxic potential.

### Statistical methods

Chi‐square and Fisher Exact tests were used to evaluate the association between categorical variables. Parametric tests (analysis of variance with Bonferroni multiple comparison post‐test) were used for cases with normal distributions and homogeneous variances, and the results were expressed as means ± standard errors of the mean (SEM). Non‐parametric tests (the Kruskal–Wallis test with Mann–Whitney *U* Test adjusted for multiple comparisons) were used for cases with non‐Gaussian distributions, and the results were expressed as median, maximum, and minimum values.

We considered exposure time as the period elapsing between start of treatment and the last administration of the drug.

Time free to the secondary malignancy (TFSM) was calculated by the Kaplan–Meier product‐limit method since date of therapy start, or diagnosis for no therapy patients until development of SM or last FU: log‐rank test was used to assess differences between subgroups. Hazard ratios (HR) and 95% confidence intervals (95% CI) were estimated for each variable using the Cox univariate model. A multivariate Cox proportional hazard model was developed using stepwise regression (forward selection, enter/remove limits *P* = 0.10 and *P* = 0.15, respectively), in order to identify independent factors for outcome; potential interactions between significant variables were taken into account when developing the multivariate model. The SPSS^®^ (21.0) statistical program was used for all analyses.

## Results

From the initial population of 1144, we excluded patients who received a diagnosis of malignancies before the ET diagnosis and those with no available data about the development of a secondary malignancy (110 patients).

The median age of the remaining 1026 evaluable patients at diagnosis was 62 years (17–96); the female sex was prevalent in our cohort (females 634; males 392). In all, 681/1026 patients (66%) were tested for JAK2V617F and 59% (404/681) resulted positive, whereas 41% (277/681) resulted wild type.

As regard adjunctive risk factors, we decided to evaluate the presence of smoke, hypertension, dyslipidemia, and diabetes. At least one risk factor was observed in 42% (433/1026) of patients, with 22% (218/1026) having more than one.

Overall, hypertension was present in 43% (441/1026) of patients, smoke in 25% (257/1026), dyslipidemia in 15% (156/1026), and diabetes in 7% (73/1026). In all, 288/1026 patients (28%) did not present adjunctive risk factors, whereas for 8% of our population this datum was missing.

Median follow‐up of the entire population was 6.2 years (0.1–32); 128 patients had a follow‐up of >15 years, 40 of >20, and 3 of >30. In all, 63/1026 patients (6%) developed 64 SM, at a median time of 4.8 years (range: 0–21.7). Type of SMs is shown in Table 1. The most frequent neoplasms (n 15, 23%) were the genitourinary ones: 7 prostate, 4 bladder, 2 kidney, 1 ovary, and 1 uterus carcinomas. Then, we found 22% of breast, 17% of lung, and 12.5% of gastrointestinal cancer. Focusing on sex‐related type of cancer, we observed that 7/392 (1.8%) males, suffered of prostate cancer [median age: 63 years (range: 55–81); 6 treated with HU and 1 with ALK] and that 13/634 females (2.1%) suffered of breast cancer [median age: 63 years (range: 38–78), eight treated with HU, two with HU and ALK, and three not treated at all]. Five patients developed a hematologic malignancy other than myelodysplastic syndrome (MDS) or AML. In particular, we observed two cases of chronic lymphocytic leukemia (CLL), two cases of non‐Hodgkin lymphomas (NHL) and one case of multiple myeloma (MM).

**Table 1:** Types of secondary malignancies

Genito‐urinary	15 (23%)
Breast	14 (22%)
Lung	11 (17%)
Gastro‐intestinal	8 (12.5%)
Hematologic	5 (8%)
Skin cancer other than basalioma	5 (8%)
Thyroid	3 (5%)
Central nervous system	1 (1.5%)
Soft tissue	1 (1.5%)
Unknown	1 (1.5%)

The median time to the development of the SM was 112 months (31–341) for skin tumors, 79 (2–193) for genitourinary, 54 (2–139) for breast, 45.5 (19–260) for gastrointestinal, 50 (2–96) for lung, and 86 (70–251) for hematologic cancers. As it is shown in Table 2, the majority of patients was treated with HU alone (62.5% of the entire cohort). Moreover, 21.5% of patients had never been treated with cytoreductive therapy.

**Table 2:** Patients distribution in different treatment groups

Group	Number of patients	%
No therapy	220	21.5
HU	641	62.5
ALK	26	2.5
ALK + HU	86	8.5
ANA/IFN	53	5

HU, hydroxyurea; ALK, alkylating agents; ANA, anagrelide; IFN, *α*‐interferon.

We did not find statistically significant differences in sex distribution in the different treatment groups (*P* = 0.22). On the contrary, we found a statistically significant difference (*P* < 0.0001) as regard age distribution in the different treatment groups. In particular, in the “no therapy” and “ANA/IFN” groups, the most part of patients were <60 year old.

**Figure 1:**
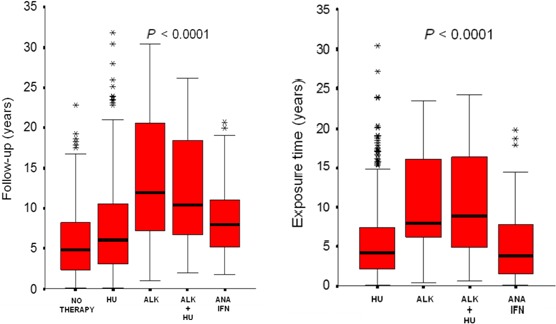
Follow‐up and exposure time in the different therapy groups.

We could also find a statistically significant difference as regard follow‐up (*P* < 0.0001) and exposure time (*P* < 0.0001) (Fig. 1) in the different treatment groups. In particular, median follow‐up was as follows: “no therapy,” 5 years (range: 0.1–23); “HU,” 6 years (range: 0.1–32); “ALK,” 12 years (range: 1–30); “ALK + HU,” 10 years (range: 2–26); “ANA/IFN,” 8 years 2, 3, 4, 5, 6, 7, 8, 9, 10, 11, 12, 13, 14, 15, 16, 17, 18, 19, 20, 21. Median exposure time to drug was as follows: “HU,” 4 years (0.1–30); “ALK,” 8 years (0.4–23); “ALK + HU,” 9 years (0.6–24); “ANA/IFN,” 4 years (0.1–20). With regard to the exposure time to drugs, in the subgroups analysis, a statistically significant difference was found between the groups “HU” vs “ALK” (*P* = 0.036), “HU” vs “ALK+HU” (*P* = 0.006), and “ALK + HU” versus “ANA/IFN” (*P* = 0.006).

As regard the cumulative rate of SM, we did not find a significant difference among the treatment groups (*P* = 0.76), irrespective of the different follow‐up and exposure time as previously mentioned. In particular, SM occurred in 5% of patients in the “no therapy” group, in 7% of patients in the “HU” group, in 8% of patients in the “ALK” group, in 8% of patients in the “ALK + HU,” and in 4% of patients in the “ANA/IFN” group (Table 3). Moreover, we looked for difference in terms of SM‐free survival between treated versus no treated patients and between no treated + “ANA/IFN”‐treated patients versus “HU,” “ALK,” and “ALK/HU”‐treated patients, but we did not observe any statistically significant difference (*P* = 0.90 and *P* = 0.63, respectively).

**Table 3:** SM rate in the different treatment groups

Groups	SM No	SM Yes	FU (years) median	ET (years) median
No therapy	209 (95%)	11 (5%)	5 (0.1–23)	NA
HU	599 (93%)	42 (7%)	6 (0.1–32)	4 (0.1–30)
ALK	24 (92%)	2 (8%)	12 (1–30)	8 (0.4–23)
ALK + HU	79 (92%)	7 (8%)	10 (2–26)	9 (0.6–24)
ANA/IFN	51 (96%)	2 (4%)	8 (2–21)	4 (0.1–20)

HU, hydroxyurea; ALK, alkylating agents; ANA, anagrelide; IFN, *α*‐interferon; SM, secondary malignancy; FU, follow‐up; ET, exposure time.

**Figure 2:**
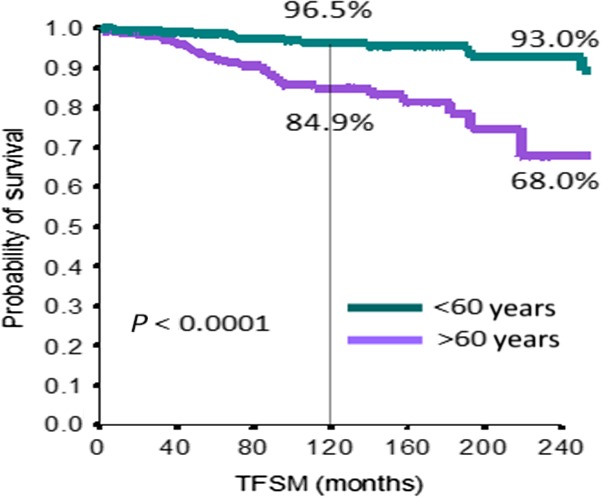
Kaplan–Mayer curve for secondary malignancy as regard as age.

**Figure 3:**
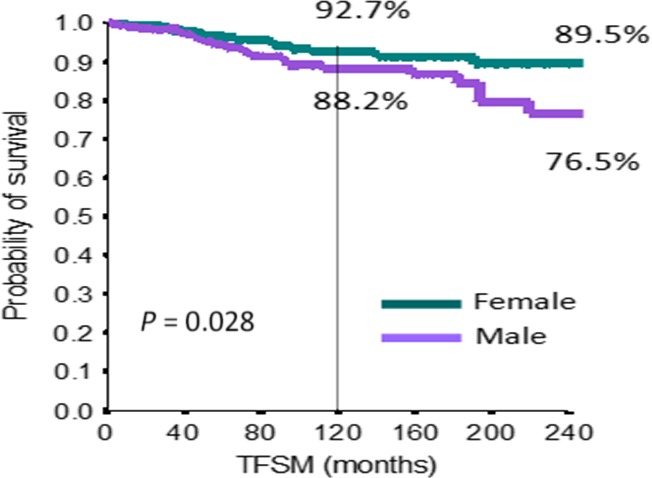
Kaplan–Mayer curve for secondary malignancy as regard as sex.

At univariate analysis, we considered exposure time to drugs, presence or absence of adjunctive risk factors, number of risk factors, different therapy groups, sex, and age (Table 4). At the multivariate analysis, the only significant prognostic factors were sex and age (HR 1.70, CI 95% 1.04–2.82, *P* = 0.03 and HR 4.19, CI 95% 2.32–7.61, *P* < 0.0001, respectively) (Table 4). Figures 2 and 3 show the Kaplan–Mayer curves: 10‐year TFSM was 96.5% for patients <60 years, while it was 84.9% for patients >60 years (*P* < 0.0001); 10‐year TFSM was 92.7% for women and 88.2% for male patients (*P* = 0.028).

**Table 4:** Cox hazard regression model

Variables	Univariate analysis			Multivariate analysis		
	HR	CI 95%	*P*	HR	CI 95%	*P*
Exposure time (<5 vs. >5 year)	1.59	0.94–2.69	0.09	–	–	ns
Adjunctive risk factors (yes vs. no)	1.04	0.59–1.80	0.90	–	–	ns
Number of risk factors	–	–	0.81	–	–	ns
(1 vs. 0)	1.12	0.62–2.02	0.72			
(>1 vs. 0)	0.90	0.44–1.85	0.78			
Therapy	–	–	0.56	–	–	ns
HU versus no	1.20	0.62–2.34	0.59			
ALK versus no	0.53	0.11–2.45	0.41			
ALK+HU versus no	0.73	0.27–1.92	0.52			
ANA/IFN versus no	0.64	0.14–2.88	0.56			
Sex (male vs. female)	1.74	1.06–2.87	0.03	1.7	1.04–2.82	0.03
Age (>60 vs. <60 years)	4.21	2.32–7.64	<0.0001	4.19	2.32–7.61	<0.0001

## Discussion

In our retrospective series of 1026 ET patients diagnosed and followed during a 30‐year period, the different therapies administered, including HU and ALK, do not appear to impact on the development of SM. A similar rate of SM was in fact observed also in untreated patients. Male gender and age >60 years were the only two significant independent prognostic risk factors. It is worth of note that in the “no therapy” group which had the shorter follow‐up (Table 3), the rate was already similar to the other groups.

The increased risk of developing secondary cancer for patients with MPNs has been explored by different authors, and by different perspective. In fact, some authors, as we did, looked for an association with ET therapies; some others looked for data which could explore a genetic instability of myeloproliferative neoplasms (MPNs) increasing per se a susceptibility for the development of SM. Moreover, the majority of the studies considered as SM also the development of MDS or AL, which instead could be considered at least in part, a natural evolution of MPNs. This is why we decided to explore the association just with tumors other than MDS/AL development.

Frederiksen et al. 24 evaluated the risk of developing a subsequent cancer among patients with different types of chronic MPNs compared with the general population on a large scale, using data from the Danish National registry of patients and the Danish Cancer Registry. They found a standardized incidence ratio (SIR) for developing a non‐hematologic cancer of 1.2 (95% CI: 1.0–1.4), and for developing a hematologic cancer of 5 (95% CI: 3.6–6.9). The authors concluded that, overall, patients were at increased risk of developing both new hematologic and non‐hematologic malignancies.

In response to this article, Susini et al. 25 reported their experience on a series of 733 MPN patients [302 PV, 375 ET, 56 Idiopathic Myelofibrosis (MFI)]. Their results showed the absence of a specific pattern of risk of cancer of all site (SIR = 0.87, 95% CI: 0.64–1.14) except for melanoma cases. So these authors suggested that the low SIR value found in the Danish study together with their negative results should call for much caution before accepting “d'emblee” the idea that the incidence of non‐hematologic cancers is specifically increased in patients with MPNs.

As regard the association with treatment, Finazzi et al. 26, in their prospective follow‐up study, explored the incidence of secondary leukemia and other malignancies in 114 ET patients who had received HU, with or without busulfan (BU), or no chemotherapy at all over a median follow‐up period of 73 months. The difference in cancer‐free survival was statistically significant between the HU + BU versus the untreated group (*P* < 0.0001) but not between the HU alone and the untreated group. However, the size of different groups was small and SMs were considered together with MDS/AL.

Radaelli et al. 18, retrospectively, investigated long‐term development of hematologic and non‐hematologic SM in 331 ET patients, analyzing possible association with chemotherapy treatments. The results of the analysis showed that treatment of ET patients with ALK was associated with a significantly increased risk of developing second hematologic malignancy (*P* = 0.03), while no association with treatment was observed as to the risk of developing non‐hematologic malignancy (*P* = ns). These results are in agreement with ours, as shown in Table 3.

Masarova et al. 27 measured the prevalence of SM in 417 patients (168 with ET, 249 with PV) and analyzed possible association with treatment received. The cumulative incidence of SM was 7.7%. Although the SIRs implied an increased probability for these patients to develop a SM compared to the general population in the United States, the only statistically significant results related to the occurrence of NHL, likely because fewer than five cases were observed in the other SM types. Surprisingly, they observed a significantly higher number of SM in patients who had received no prior therapy (17%), as compared to patients who had received monotherapy (9.6%), or multiple therapies (4%) (*P* = 0.003).

In recent years, several studies highlighted that Philadelphia‐negative MPNs are associated with an increased risk of chronic inflammation diseases, autoimmune diseases, and second cancer as well, not directly associated with treatment 24, 28. Furthermore, it has been hypothesized that chronic inflammation may be a trigger and driver of second cancer in MPNs 29. In this prospective, the MPNs have also been described as “a human inflammation model for cancer development” 30, as the MPNs per se generate inflammatory products. Considering the above and general contention that chronic inflammation may be a major contributing factor for cancer development and progression, the reports on an increased risk of secondary cancer in MPNs are only supportive of a link between chronic inflammation and development of cancer in patients with MPNs as well 29, 30, 31, 32, 33. Therefore, chronic inflammation and immune deregulation in MPNs can be major contributing factors for the massive comorbidity burden 29, 30, 33 which also includes the risk of second cancer, both hematologic and non‐hematologic 24, 34, 35, 36. This concept could explain the similar rate of SM in MPNs patients treated either with or without cytoreductive treatments, and in patients not treated at all, as reported in our and other studies 18, 27.

In conclusion, data regarding the development of SM in MPNs are accumulating although the most part of results derive from retrospective studies. Moreover, the analyses usually include also “older” drugs such as alkylating agents (busulfan, pipobroman, and radio‐active phosphorus). In this context, the strength of our study resides in the large population of ET patients included and in the long follow‐up. All published data support the idea that patients with MPNs develop SM regardless of the treatment modalities given. This could be due to the intrinsic chronic inflammation and immune deregulation in MPNs which can be major contributing factors for the risk of second cancer 29, 30, 33. With this in mind, some authors suggest that it is time to rethink the goals of therapy of MPNs, when considering the rapidly accumulating and compelling evidence that platelets are deeply involved in tumor progression and metastasis. In light of these novel data, the active treatment of low‐risk patients could be considered a better option than a “wait and watch” strategy 20. A tailored approach is always mandatory, considering that the most important step for the therapeutic decision in ET patients, after an accurate diagnosis, is the risk assessment based on medical history, clinical assessments, and laboratory examination 37.

## Conflict of Interest

None.
